# PReS-FINAL-2337: The eurofever registry: 3 years of enrollment

**DOI:** 10.1186/1546-0096-11-S2-P327

**Published:** 2013-12-05

**Authors:** S Federici, J Frenkel, S Ozen, J Antòn, JI Arostegui, F De Benedetti, P Dolezalova, H Girschick, V Hentgen, M Hofer, H Lachmann, I Koné-Paut, J Kuemmerle-Deschner, B Neven, H Ozdogan, C Rose, A Simon, S Stojanov, N Toplak, I Touitou, R Vesely, P Woo, C Wouters, N Ruperto, A Martini, M Gattorno

**Affiliations:** 1 for PRINTO and Eurofever Project, Genoa, Italy; 2 Gaslini Institute, Genoa, Italy

## Introduction

The main limitation to a better knowledge of Autoinflammatory diseases is related to the extreme fragmentation of the diagnosed cases that are spread over different centers and countries. The general aim of the Eurofever Project (agreement n 2007332, EAHC) is to build an international registry on Autoinflammatory diseases.

## Objectives

To evaluate the number of patients enrolled in the registry in the first 36 months after starting the enrolment.

## Methods

A web-based registry collecting baseline and cross-sectional clinical information on Autoinflammatory diseases is available in the member area of the PRINTO web-site (http://www.printo.it). The registry is open to all pediatric and adult Centers with a specific interest in Autoinflammatory diseases. The following monogenic autoinflammatory diseases were considered: Familial Mediterranean Fever (FMF), Cryopyrin-associated periodic syndromes (CAPS), TNF receptor-associated periodic syndrome (TRAPS), mevalonate kinase deficiency (MKD), Blau syndrome, pyogenic arthritis, pioderma and acne (PAPA) syndrome, deficiency of IL-1 receptor antagonist (DIRA), NLRP12-mediated periodic fever. Information on CRMO, Behçet's disease, PFAPA and undefined periodic fevers were also collected.

## Results

2721 patients, from 221 centers in 56 countries, have been enrolled in the registry during the first 36 months. Baseline demographic data (country of residence, disease onset, disease duration, mutations, family history ect) from all patients are now available. In 2213 (81%) complete information on clinical manifestations and responses to treatments is also available. The disease distribution of enrolled patients is: FMF 787 (621 with complete clinical data); TRAPS 237 (211 with complete clinical data); CAPS 207 (186 with complete clinical data); MKD 153 (133 with complete clinical data); Blau syndrome 62 (21 with complete clinical data); PAPA 19 (18 with complete clinical data); NLRP-12 mediated periodic fever 8 (6 with complete clinical data); DIRA and Majeed 3 and 2 patients, respectively (all with complete clinical data). Among multifactorial autoinflammatory diseases: PFAPA 564 (402 with complete clinical data); CRMO 392 (370 with complete clinical data); pediatric Behçet disease 84 (68 with complete clinical data) and 205 patients with undefined periodic fever (174 with complete clinical data).

## Conclusion

A large registry of patients with Autoinflammatory diseases is available and the enrolment is still ongoing. In the next months novel inherited autoinflammatory diseases (CANDLE, DITRA) will be included and data collection will become longitudinal.

## Disclosure of interest

None declared.

